# Optical coherence viscometry

**DOI:** 10.1063/5.0048608

**Published:** 2021-04-20

**Authors:** Hsiao-Chuan Liu, Matthew W. Urban

**Affiliations:** 1 Department of Radiology, Mayo Clinic, Rochester, Minnesota 55905, USA; 2 Department of Physiology and Biomedical Engineering, Mayo Clinic, Rochester, Minnesota 55905, USA

## Abstract

We report a technique, named optical coherence viscometry (OCV), to measure the viscosity of Newtonian fluids in a noncontact manner. According to linear wave theory with small amplitudes, capillary waves are associated with fluid mechanical properties. To perform this measurement and avoid the overdamped effects of capillary waves in viscous fluids, transient acoustic radiation force was applied to generate capillary waves. Within a very limited field-of-view using optical coherence tomography, wave motion acquired in the time domain was analyzed using Fourier methods to study the wave velocity dispersion and attenuation relationships for capillary waves, which can reduce the fluid quantity drastically into tissue culture scale. We measure the viscosities of water, water–glycerol solutions with three concentrations, and biological plasma using the proposed OCV and compare the experimental results to theoretical calculations. OCV is sensitive to wave perturbations and can be a promising technique for measuring the viscosity of biological fluids and could be applied in future applications for measurements for lipid membranes in cell biology and tissue engineering investigation.

The conventional rotational rheometry method uses a horizontal plane that applies weak torque to a liquid sample to measure fluid viscosity,^1^ which needs to contact samples or mediums. This contact could have an adverse effect on biological fluids. The viscometer, another traditional technique, requires pouring a known volume of fluids in a U-shaped glass tube with a small diameter, and viscosity is associated with the flowing time of samples thoroughly a tube.^2^ Microfluidic-based methods are a relatively attractive modality for determining liquid viscosity.^3–6^ However, complicated fabrications with specific channel scales on polydimethylsiloxane (PDMS), extra dye agents, biological compatibilities, and medium compositions need to be considered.^7^

Surface waves with wavelengths of millimeters propagating along the two-phase boundary between fluid and air are known as capillary waves, which are produced by small stress exerted on the surface of fluids.^8^ Harmonic excitation is a common method to produce capillary waves. However, it becomes difficult to study capillary waves in viscous fluids due to overdamped effects.^9^ Using a large tank provides extended distance between the excitation source with a specific low frequency (e.g., ∼150 Hz for 20% glycerol–water solution) and detector to avoid the effect of overdamped capillary waves.^10^

By applying an electric field, capillary waves can be produced with a noncontact approach.^11^ However, a controlled environment is required to avoid mechanical disturbances and air currents which could damage cellular or molecular components in biological media. Methods using light scatter by thermal excitation to produce capillary waves show unreliable rheological results.^10,12^ Optical coherence tomography (OCT) has been used to measure mechanical properties in solid materials over the last decade.^13–15^ OCT was proposed for a few rheological applications to observe the velocity profiles in flowing fluids with added suspension particles.^16,17^ However, the aforementioned methods may have limited use for biological applications.

In this Letter, we report a rheological modality, optical coherence viscometry (OCV), to measure the viscosity of Newtonian fluids in a noncontact manner. The OCV combines transient acoustic radiation force and OCT to evaluate the viscosity of fluids via capillary waves. For rheological measurements, previous approaches to study capillary waves in viscous fluids required volumes in a large tank to avoid overdamping effects. Using transient acoustic radiation forces to produce capillary waves could avoid this problem^9^ and can be applied to various fluids without specifying a frequency; consequently, the phase velocity of capillary waves can be measured within a very limited field-of-view in OCV, which can reduce the fluid quantity drastically and could approach tissue culture scale for applications in biological fields.

According to Navier–Stokes theory,^18^ the continuity equation in fluids to describe the flow velocity vector field $\vec{v}$ is given by

$$\frac{\partial \rho }{\partial t}+\nabla \;\cdot \;(\rho \vec{v})=0,$$ (1)

where *ρ* is fluid density and *t* is time. Assuming a fluid-air free surface with infinitesimal amplitude so that the linearized approximation suffices and fluid with uniform depth, irrotational, and incompressible with constant *ρ*, the continuity equation is simplified as

$$\nabla \;\cdot \;\vec{v}=0.$$ (2)

Assuming capillary waves propagate in the *x*-direction at a mean surface elevation (*z *=* *0) during the time *t*, the velocity potential Φ(*x*, *z*, *t*) can be written as the vector component of flow velocity $v_{x}$ in *x* and $v_{z}$ in *z* so that

$$\vec{v}=\nabla \Phi .$$ (3)

Substituting Eq. (3) into (2), the Φ(*x*, *z*, *t*) must satisfy the Laplace equation and can be written as

$$\nabla^{2}\Phi =0.$$ (4)

Considering waves are cyclic movements with time and horizontal position, *x*, and vary with depth *z*, we can simply assume that

$$\Phi \left(x,z,t\right)=\xi (z)e^{i(kx-\omega t)}=\;\xi (z)e^{ik(x-c_{p}t)},$$ (5)

where ξ is wave amplitude, *k* is the wavenumber, *x* is the wave traveling direction, and *c_p_* is phase velocity of waves at angular frequency *ω* = 2*πf*. Substituting Eq. (5) into (4), we obtain

$$\frac{\partial^{2}\xi }{\partial z^{2}}+\xi k^{2}=0.$$ (6)

If the distance *h* at the impermeable bed underneath the fluid layer is constant, the boundary condition on the bed is given by

$$v_{z}={\left.\frac{\partial \Phi }{\partial z}\right|}_{z=-h}=0,$$ (7)

so that $\Phi \left(x,z,t\right)$ can be expressed as

$$\Phi \left(x,z,t\right)=\gamma \;\cosh\;k(z+h)\;e^{ik\left(x-c_{p}t\right)},$$ (8)

where γ is an arbitrary constant and γcoshk(z+h) is defined as ξ(z).

Due to the assumption of the infinitesimal waves on a free surface, the kinematic free surface boundary condition must satisfy

$${\left.\frac{\partial \xi }{\partial z}=\frac{\partial \varepsilon }{\partial t}\right|}_{z=\varepsilon (x,\;t)},$$ (9)

where ε(x, t) is instantaneous surface wave elevation and can be described as

$$\varepsilon \left(x,y\right)={\eta e}^{ik(x-c_{p}t)},$$ (10)

where η is the wave elevation. Considering ε is very small to let *z *=* *0, γ is given by $ic_{p}\eta /\sinh\;kh$.

The dynamic pressure due to $\Phi \left(x,z,t\right)$ must satisfy the time-dependent Bernoulli equation. Due to small amplitude waves, curvature of the fluid surface must be considered for surface tension and the nonlinear terms can be neglected. We have

$${\left.\frac{\partial \Phi }{\partial t}+g\varepsilon +\frac{p_{0}+p_{\mathrm{sur}}}{\Delta \rho }=0\right|}_{z=\varepsilon (x,\;t)},$$ (11)

where *g* is gravity, Δρ is difference of density between the fluid and air, $p_{0}$ is surface gauge pressure, and the pressure difference across the fluid interface $p_{\mathrm{sur}}$ is a function of curvature. Because pressure is uniform on the wave surface, $p_{0}$ = 0 and $p_{\mathrm{sur}}$ is approximately to be −$\sigma \frac{d^{2}\varepsilon }{dx^{2}}$ according to the Young–Laplace equation, where σ is the surface tension. Substituting Eqs. (5) and (10) into (11), the phase velocity $c_{p}$ of capillary waves is written as

$$c_{p}=\sqrt{\left(\frac{g}{k}+\frac{\sigma k}{\Delta \rho }\right)\tanh\left(kh\right)}.$$ (12)

For capillary waves in deep water, the wavelength λ has to be smaller than $\sqrt{\sigma /\Delta \rho g}$ defined as the critical capillary length $\lambda_{c}$, *g* is negligible, and h>0.5λ to have tanh(*kh*)* *≈ 1.^19^ Simplifying, we can rewrite Eq. (12) as

$$c_{p}≅\sqrt{\frac{\sigma k}{\Delta \rho }},$$ (13)

and the dispersion relation of capillary waves in deep water is

$$\omega =\sqrt{\frac{\sigma k^{3}}{\Delta \rho }}.$$ (14)

Using the relationship $c_{g}≜d\omega /dk$, we obtain the group velocity $c_{g}$ = 1.5 $c_{p}$. Due to energy dissipation by viscous forces, ε decays exponentially as waves propagate in a *x* direction and can be described as $\varepsilon_{0}e^{-\alpha \Delta x}$, where $\varepsilon_{0}$ is reference amplitude, α is the attenuation, and Δx is the wave travel distance. On the fluid surface, the energy loss per unit time must be equal to the viscous power loss per unit area, and the fluid viscosity μ is given by^10^

$$\mu =\frac{\Delta \rho \alpha }{2k^{2}}{1.5c}_{p}.$$ (15)

According to Eq. (15), viscosity is strongly related to wave velocity, attenuation, density, and surface tension. The values of σ and ρ were used from Ref. 20 and *h* is assumed to be 8 mm, corresponding with our experiments, for subsequent calculations. The theoretical calculations of $c_{p}$, α, and μ in water, 20%, 40%, and 60% water–glycerol solutions by weights (G7757-1L, ≥99.0% Reagent Grade, Honeywell, MI, USA) were illustrated in Fig. 1. The $c_{p}$ in the water–glycerol solutions were slightly slower than in water, which is due to the viscous properties of the mixtures.^21,22^ The $c_{p}$ changed weakly with respect to the viscosity, but α had a strong sensitivity to the viscosity variation. The $\lambda_{c}$ is 17.3 mm, 16.5 mm, 15.9 mm, and 15.3 mm in water, 20%, 40%, and 60% water–glycerol solutions, respectively [Fig. 1(b)].

**FIG. 1.**
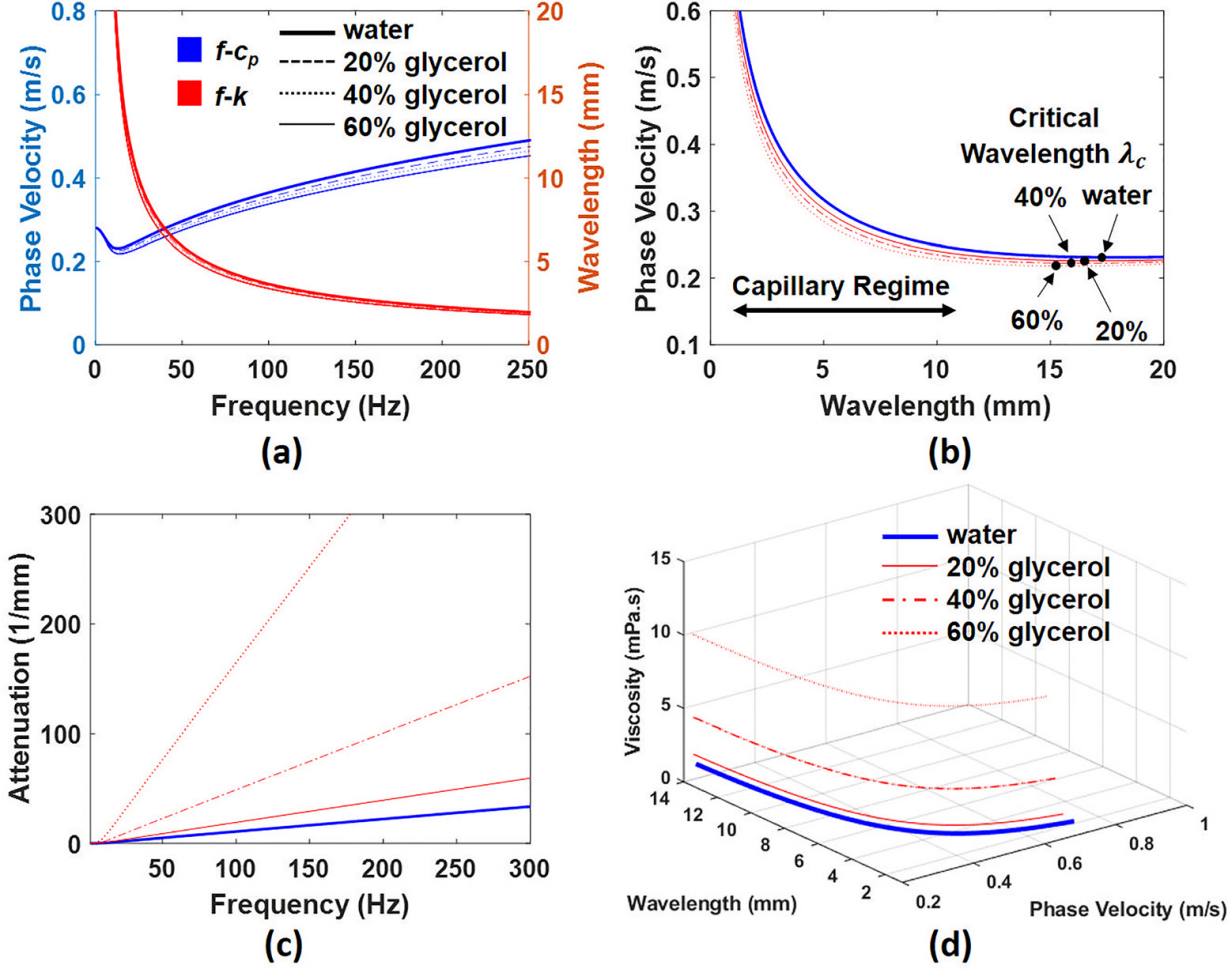
(a) The theoretical calculation shows the relationship between frequency and $c_{p}$ (blue) and between frequency and *k* (red) by Eq. (12). (b) shows the $c_{p}$ functions of *k* and illustrates the ${\;\lambda }_{c}$ in water, 20%, 40%, and 60% water–glycerol solutions. The α and μ are presented in (c) and (d) by Eq. (15), respectively.

Experimentally, the system structure of OCV is illustrated in Fig. 2. The spectral-domain optical coherence tomography (SD-OCT) system (TEL320C1, Thorlabs Inc., Newton, NJ, USA) is equipped with a 1300 nm source and LK4 lens kit (Thorlabs Inc., Newton, NJ, USA) to provide a 16 mm × 16 mm field of view. A 10 kHz A-scan rate was selected to track capillary wave perturbations in the space-time domain. The customized two-dimensional (2D) acquisition was set as 640 axial scans to provide a 25 *μ*m lateral spacing over 16 mm and 500 repeated scans at each lateral location over a 50 ms time span (M-B scan). Each data point includes a real and imaginary value from which magnitude and phase information can be obtained. Three function generators (33500B, Keysight, Santa Rosa, USA) are used to synchronize the acoustic radiation force excitation with the OCT system and produce the ultrasound for the acoustic radiation force push. A radio frequency (RF) amplifier is used to amplify the ultrasound signal for application to the transducer.

**FIG. 2.**
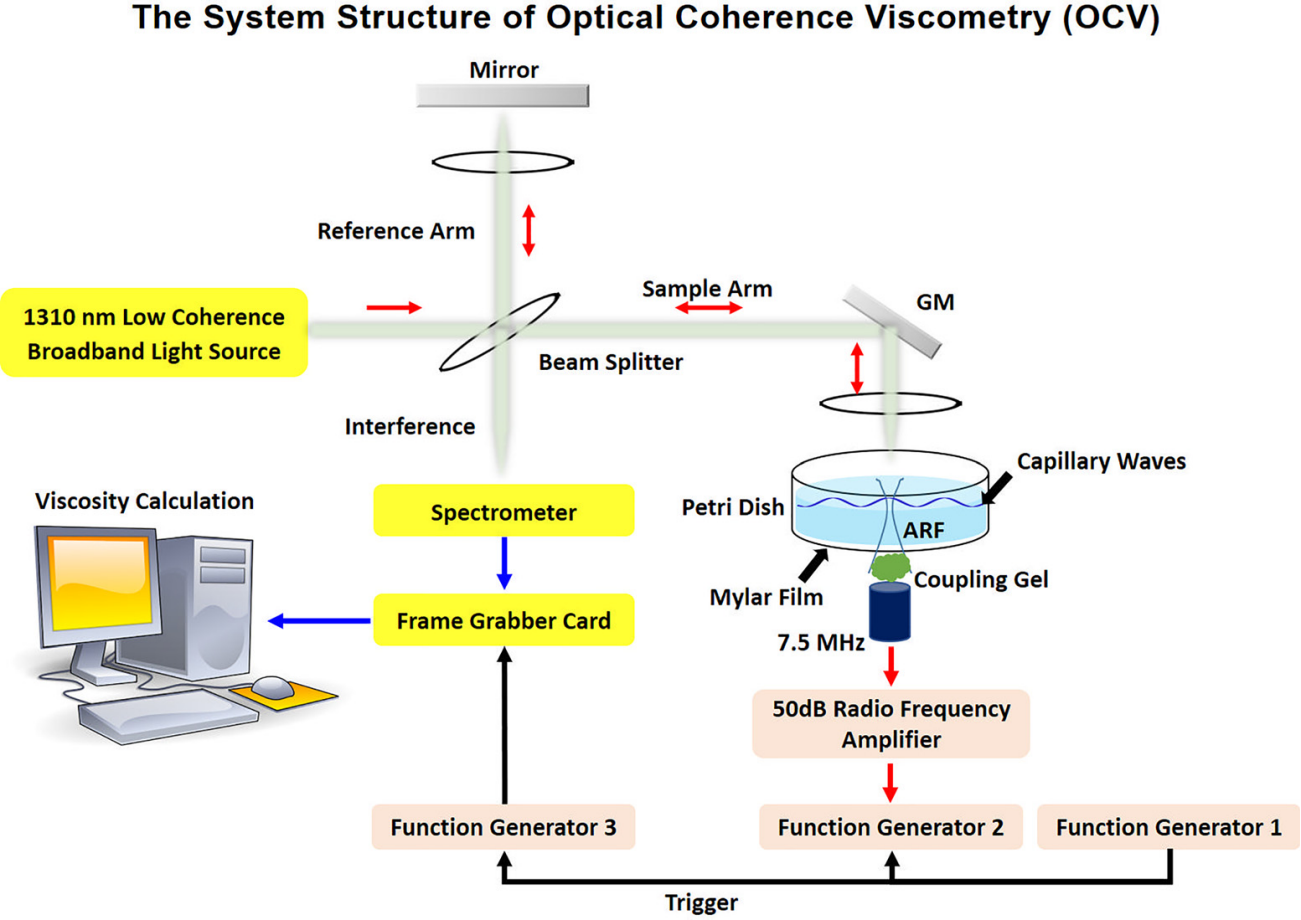
Schematic of optical coherence viscometry (OCV) acquisition system. The three function generators are utilized to provide trigger and driving signals for synchronizing the entire OCV system, excitation, and recording signals. The OCT system is shown with its components, and GM represents the galvanometer mirror.

The OCV can detect very small perturbations (<5 *μ*m) of capillary waves on the surface of fluids due to the advantage of the low coherence interference property presented in Fig. 3(a). Compared with capillary waves traveling in a larger tank,^11^ OCV is a promising modality to observe capillary waves within a 16 mm field-of-view, which can dramatically reduce the volume of fluids needed to the cell culture dish scale.

**FIG. 3.**
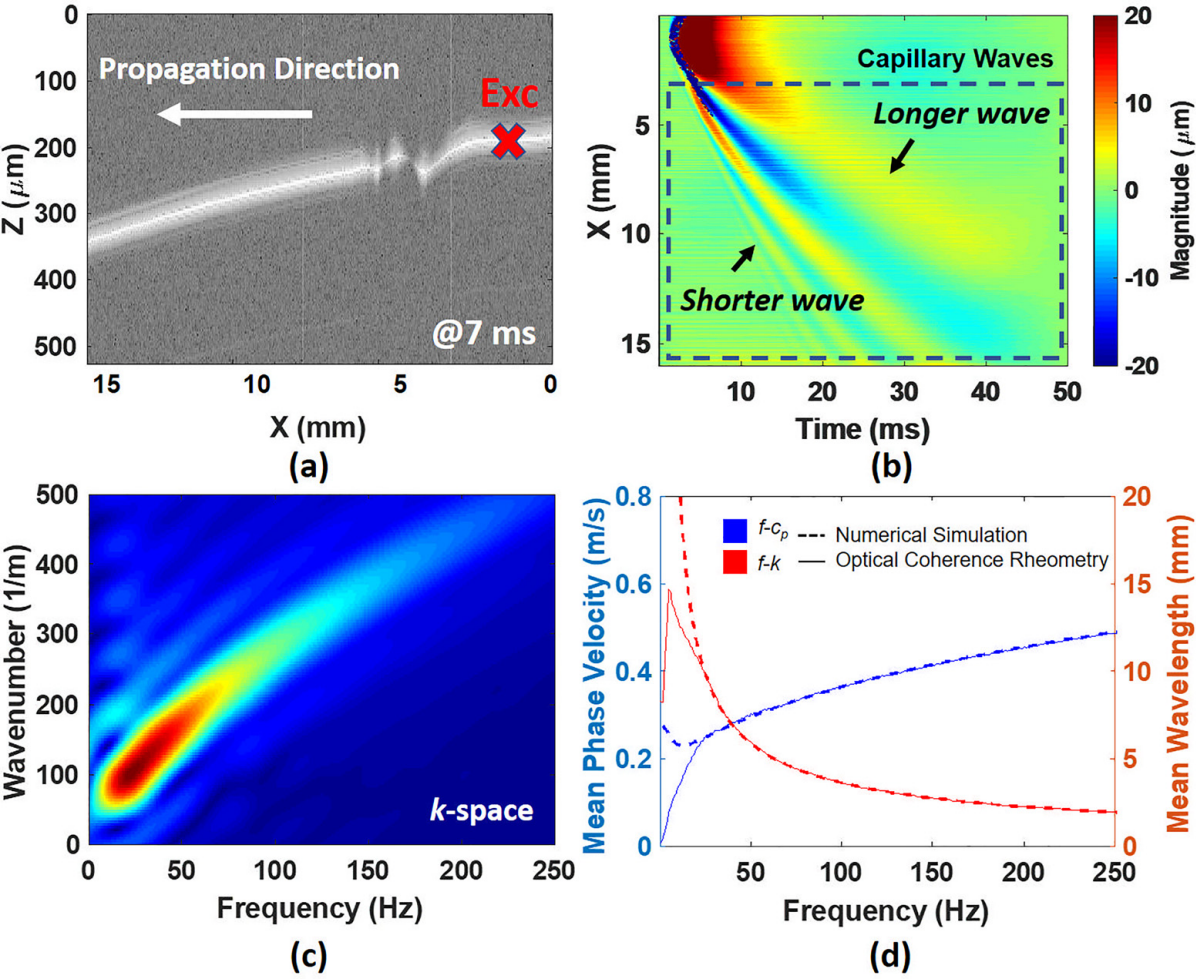
(a) The capillary wave on water was detected by OCV. (b) The spatiotemporal wave motion map from (a) was constructed by using autocorrelation. (c) A *k*-space representation was calculated using a 2D-FFT and (d) demonstrates that the $c_{p}$ for water measured by OCV is in strong agreement with theoretical calculations. Exc: excitation position.

A 7.5 MHz focused transducer (ISO703HR, Valpey-Fisher, Hopkinton, MA, USA) was used to generate acoustic radiation force (considered as a point excitation) using 500 *μ*s sinusoidal pulses. The force was exerted on the fluid–air interface to produce capillary waves by coupling the transducer to the Mylar film bottom of a customized Petri dish such that the ultrasound waves travel through the fluid and interacts with the fluid–air interface at the focal point of the transducer.^19^ In this case, there is no direct contact with the sample with the OCV system. The lateral focal size is approximately 510 *μ*m calculated using 2.44×f#×λ,^23^ which generates waves similar to the ripples on a water surface created by dropping a stone in a pond assuming hλ ≫1.^18,19^ Using acoustic radiation force and a small field-of-view, overdamping effects in viscous fluids can be avoided in OCV measurements.

A spatiotemporal wave motion map was reconstructed by autocorrelation on the complex data,^24^ presented in Fig. 3(b). A median filter was employed to remove noise from a two-dimensional wave motion image before autocorrelation was performed. The data in each column of the wave motion image were upsampled by five times using spline interpolation. After the autocorrelation calculation, the optical coherence elastography (OCE) data were averaged over depth to make the baseline of wave motions to be zero and spatiotemporal wave motion maps were reconstructed with time on the horizontal axis and propagation distance on the vertical axis. The details of the wave reconstruction method can be found in our previous reports.^19^

A two-dimensional fast Fourier transform (2D-FFT) with size 5120 × 5120 points was used to decompose multiple frequencies from spatiotemporal wave motion map into a Fourier representation that has spatial and temporal components (*k*, *f*), which has been termed as *k*-space [Fig. 3(c)].^25^ From the *k*-space, the $c_{p}\;(≜2\pi f/k$) and λ curves vs frequency are determined and shown in Fig. 3(d). Ten replicate measurements were performed for water and water–glycerol solutions, while 20 measurements were made for porcine plasma (LAMPIRE Biological Laboratories, PA, USA). The detailed protocol of plasma preparation can be found in Ref. 19. The experiments were carried out at ambient temperature (22 ± 1 °C) measured by a thermometer (TMD-56, Amprobe, WA).

The wave amplitude ε exponentially decays as waves propagate in the *x* direction due to energy dissipation by viscous forces.^10^ Hence, the accuracy of estimating α largely depends on the viscosity of fluids. Based on the spectral analysis of Fig. 3(b) by using a one-dimensional (1D) Fourier transform, we observe that the magnitude change with respect to distance becomes a smooth exponential-like trend at frequencies at 100 Hz and above, Fig. 4(a). This persists for frequencies up to 250 Hz, and then the relative magnitude decreases to a point, where accurate detection may be compromised.

**FIG. 4.**
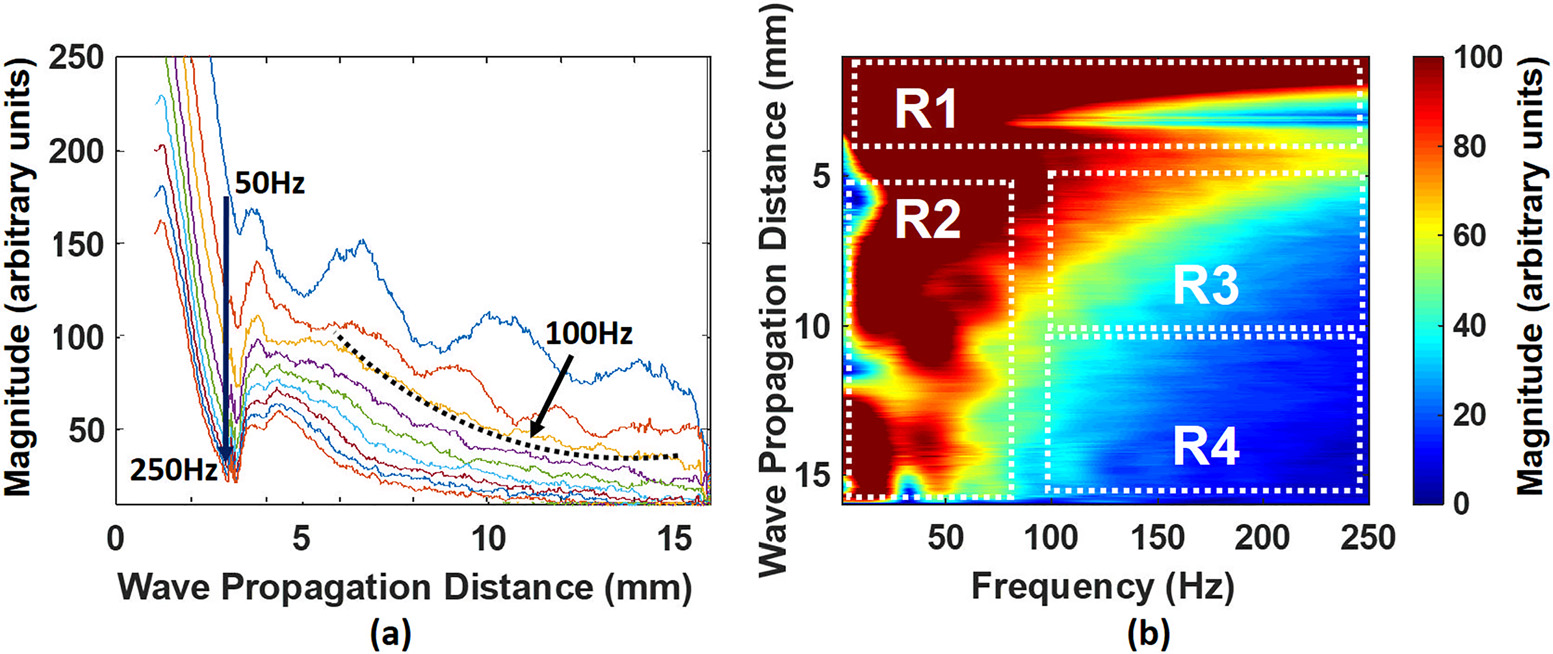
The capillary waves traveling on water at different locations were broken down into various frequency components in frequency domain. (a) Determining frequency range, and (b) determining traveling distance of capillary waves for α. R: region.

Figure 4(b) illustrates the magnitude of components at different frequencies vs wave propagation distance for the water case. The regions R1 and R2 display strong artifacts at low frequencies caused by the near field effect of the acoustic radiation force composed of the push beam location and its proximal surroundings. By evaluating the spectral information in Fig. 4(a), we determined that the range from 5 to 10 mm (R3), corresponding with both longer and shorter capillary wave regimes, was the most suitable range for evaluating α in the water case as well as the other cases studied. The R4 region is not appropriate to use for evaluating attenuation due to nearly uniform distribution of the energy after 10 mm of wave propagation. In this case, the magnitude changes could not reliably be evaluated.

The advantage of using frequency domain analysis is the improved stability and accuracy of attenuation of capillary waves for two-phase system with arbitrary fluid properties instead of conventional methods in time domain.^8,26^ In Fig. 5(a), OCV provides the attenuation result vs frequency (red solid line), which is in good agreement with the theoretical calculation (blue dash line). The viscosity of water (mean ± standard deviation) for ten repeated OCV measurements at ambient temperature was 1.06 ± 0.07 mPa s presented in Fig. 5(b), which corresponds with the results of 1.0020 mPa s from the well-known literature by Korson *et al.*^27^

**FIG. 5.**
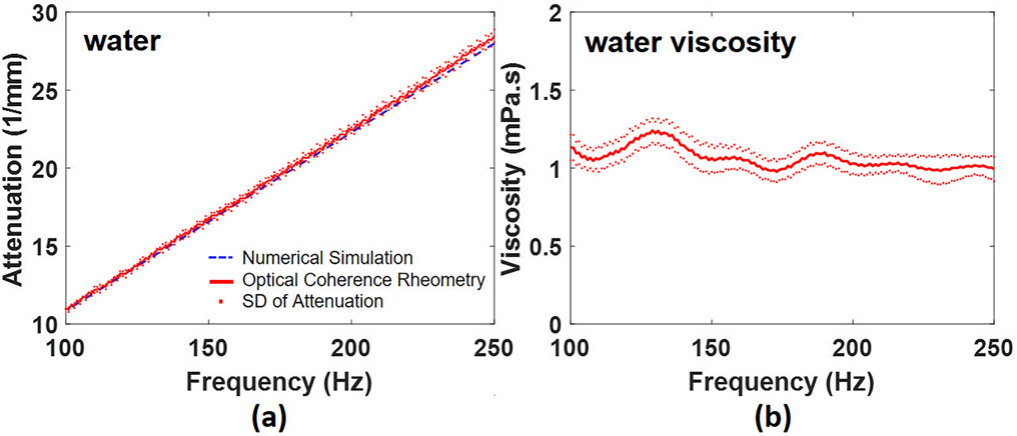
(a) The results of attenuation of capillary waves in water between OCV and theoretical calculations. (b) The viscosity of water at the frequencies from 100 Hz to 250 Hz. SD: standard deviation.

Another report noted that the aqueous viscosity ranges from 1.04 to 1.15 mPa s at 20 °C.^20^ By using typical viscometers (Model 1380FM, VWR International), the results of the measured viscosity show a 10% deviation,^20^ which demonstrates that viscosity is highly sensitive to the environment even using typical viscometers. Comparing our results with ten repeated OCV measurements at ambient temperature, the presented results from this study are very consistent and in agreement with other literature values.

We demonstrate that $c_{p}$ and λ obtained by OCV are in good agreement with theoretical calculations in 20%, 40%, and 60% water–glycerol solutions, illustrated in Fig. 6. As we mentioned before, $c_{p}$ is not highly sensitive to *μ*. In OCV, we observe that measured $c_{p}$ matches theoretical calculations at lower frequency ranges as the viscosity of fluids is gradually increased, which is reasonable due to energy dissipation by viscous forces. In other words, fluids with lower viscosities can use a wider frequency range to estimate α.

**FIG. 6.**
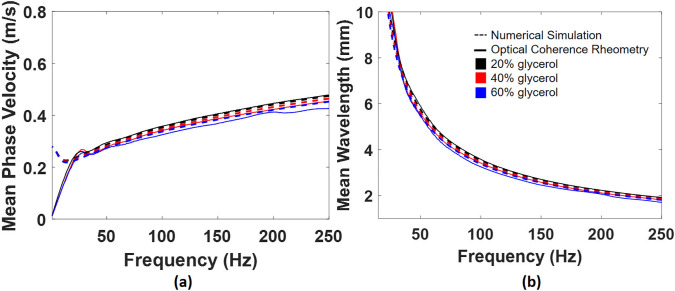
The comparison between OCV and theoretical calculations of (a) $c_{p}$ and (b) λ vs frequency in 20%, 40%, and 60% water–glycerol solutions.

In Fig. 7(a), we obtained α in 20%, 40%, and 60% water–glycerol solutions by OCV. To compare them with their theoretical calculations, the α is highly consistent with the theory in specific frequency ranges. Based on the spectral analysis, we observe that the frequency from 50 to 150 Hz is a reliable range to estimate attenuation of capillary waves for 20% water–glycerol solutions. However, only the frequency range from 15 to 20 Hz can be used in 60% water–glycerol solutions, which could be a limitation of OCV due to strong attenuation.

**FIG. 7.**
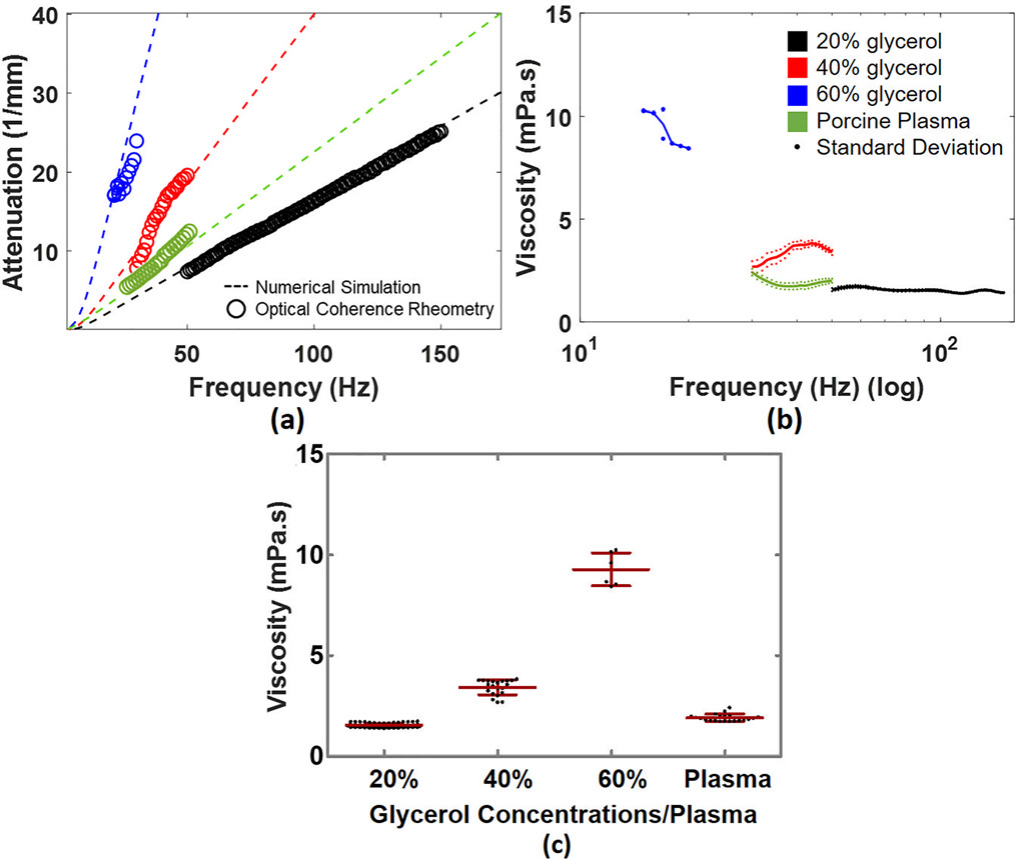
(a) Shows the attenuation in 20%, 40%, 60% water–glycerol solutions and porcine plasma between OCV and theoretical calculations. (b) The viscosity vs frequency was demonstrated in four viscous fluids. (c) A scatter dot plot illustrates the mean viscosity and standard deviation in four different viscous fluids.

Based on ten repeated measurements of water–glycerol solutions, we demonstrate that the viscosity (mean ± standard deviation) of 20%, 40%, and 60% water–glycerol solutions at ambient temperature were 1.53 ± 0.08 mPa s, 3.42 ± 0.38 mPa s, and 9.26 ± 0.84 mPa s, respectively, illustrated in Figs. 7(b) and 7(c). A well-known report published by Takamura *et al.* described that the viscosity of water–glycerol solutions in the temperature around 20–30 °C approximately from 1.80 to 1.50 mPa s in 20%, from 3.90 to 3.00 mPa s in 40%, and from 12.00 to 8.00 mPa s in 60%.^20^ Another well-known article by Cheng^28^ demonstrates that the viscosity of water–glycerol solutions at the temperature 22 °C is 1.65 mPa s, 3.46 mPa s in 40% and 10.02 mPa s in 60% by using the website tool.^29^ We demonstrate a good agreement by using OCV with reported results. We also demonstrate that OCV was applied to porcine plasma at ambient temperature and obtained viscosity of 1.90 ± 0.18 mPa s presented in Figs. 7(b) and 7(c). According to Duck,^30^ the viscosity of plasma ranges from 1.67 to 2.35 mPa s, which is consistent with our values obtained using OCV.

The standard deviations of the viscosity values are of the order of approximately 5%–10% in the study by using OCV, 6.6% for water 5.2% for 20% glycerol, 11% for 40% glycerol, 9% for 60% glycerol, and 9.4% for porcine plasma. Comparing this to data from a commercial viscometer,^20^ the order of precision ranges was reported from 3 to 9%, 4.2% for water, 3.1% for 20% glycerol, 8.6% for 40% glycerol, and 6% for 60% glycerol. It is possible to obtain better precision by improving the algorithm of the attenuation calculation in the future; however, the precision of OCV seems to be promising in the study.

The results presented above demonstrate that OCV, a rheological technology for measuring viscosity, can robustly be applied to fluids in a noncontact manner. The capillary waves can be monitored in a 16 mm field-of-view so that the necessary volume of media potentially reduces to the cell culture dish scale. In addition, using transient acoustic radiation forces as externally applied forces to produce capillary waves could reduce the measurement distance from the excitation source, compared with harmonic excitation which requires longer distances to avoid damping of capillary waves in viscous fluids. Compared with thermally or electrically excitation as the noncontact methods, transient acoustic radiation force should not damage cells in biological media.

Although our OCV system has not been used to test viscosity calibration standards (typically oils), it has been reported that the glycerol can also be used to calibrate viscosity.^31^ Glycerol solutions may not be entirely suitable liquids for use as viscosity standards in calibrating viscometers mainly because of hygroscopic properties; however, it would not be an issue if precautions are taken to obviate deviations in concentrations.^32^ The viscosity standards will be considered to calibrate OCV in the future study.

Due to the advantage of the low coherence interference property of OCV, small wave perturbations on the fluid surface can be used to evaluate the wave attenuation accurately. The mean viscosity of human body fluids ranges from 1.2 to 6.45 mPa s.^30^ In this Letter, we demonstrate OCV is able to provide the measurement of viscosity of Newtonian fluids up to approximately 10 mPa s. The previous literature reported that the properties of polymer solutions and lipid membranes can be evaluated with capillary waves.^8,33^ In the future, studies examining the relationship between wave velocity and attenuation with respect to shear rate will be investigated to determine viscosity in non-Newtonian fluids. In addition, an accurate temperature controller will be placed on a Petri dish for our future applications in tissue engineering for creating an environment for keeping cells alive. With various advantages of OCV, we demonstrate that it can be a potentially promising viscometer for measuring not only the viscosity of biological fluids but also lipid membranes in tissue engineering and cell biology future applications.

## Data Availability

The data that support the findings of this study are available from the corresponding author upon reasonable request.
